# Clinical outcome following magnetic resonance imaging as first-line imaging in low-impact pediatric spine trauma: a single-center retrospective observational study

**DOI:** 10.1007/s00247-023-05721-7

**Published:** 2023-07-31

**Authors:** Aapo Sirén, Mikko Nyman, Johanna Syvänen, Kimmo Mattila, Jussi Hirvonen

**Affiliations:** 1 Department of Radiology, University of Turku and Turku University Hospital, Kiinamyllynkatu 4-8, 20520 Turku, Finland; 2 Department of Pediatric Orthopedic Surgery, University of Turku and Turku University Hospital, Turku, Finland; 3 Medical Imaging Center, Department of Radiology, Tampere University and Tampere University Hospital, Tampere, Finland

**Keywords:** Emergency, Magnetic resonance imaging, Pediatric, Spine, Trauma

## Abstract

**Background:**

Pediatric spinal trauma is rare, but the consequences of a missed injury can be devastating. Medical imaging is often needed in addition to physical examination. Conventional radiographs are widely recommended, but their negative predictive value is limited. Computed tomography (CT) is more sensitive but has a higher radiation dose. Magnetic resonance imaging (MRI) has superior soft tissue contrast and lacks ionizing radiation, but it is more expensive and time-consuming. Thus, the debate regarding the most suitable imaging method is still ongoing.

**Objective:**

This study examined the ability of MRI to exclude injuries requiring surgical treatment as a first-line imaging method in low-impact pediatric spine trauma.

**Materials and methods:**

We retrospectively reviewed the medical records and imaging data of children (under 18 years old) who had suspected spinal trauma and were scanned using MRI in our emergency radiology department over a period of 8 years. We assessed the ability of MRI to detect unstable injuries by searching for later occurrences of primarily missed injuries requiring surgery.

**Results:**

Of 396 patients (median age 12 years, range 0–17), 114 (29%) had MRI findings suggesting an acute injury. Bony injuries were detected in 78 patients (20%) and ligamentous or other soft tissue injuries in 82 patients (21%). In the subgroup of 376 patients (median age 12 years, range 0–17) with at least 6 months of clinical follow-up, no missed injuries demanding surgical intervention or immobilization occurred after spinal MRI as  first-line imaging. No adverse events related to MRI or anesthesia occurred.

**Conclusion:**

MRI can detect injuries requiring surgical treatment as a first-line imaging method in suspected low-impact pediatric spinal trauma and is safe to use in this selected population.

**Graphical abstract:**

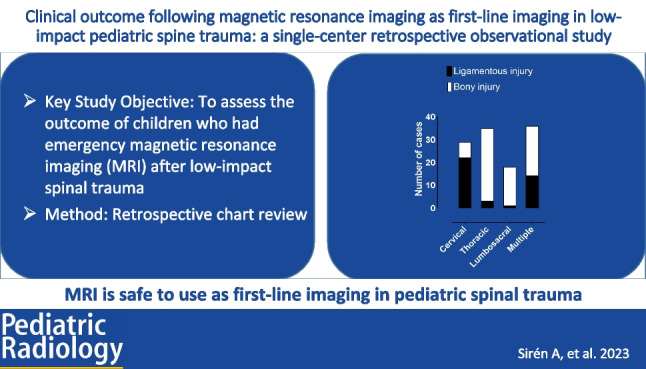

**Supplementary information:**

The online version contains supplementary material available at 10.1007/s00247-023-05721-7.

## Introduction

Pediatric spinal injuries present a diagnostic dilemma in the emergency setting. To rule out a possible spinal injury and despite numerous clinical algorithms, in many cases spinal imaging is still needed [1]. Although the exact incidence is not known [2, 3], pediatric spinal trauma is rare. In a large Finnish registry-based study, the overall incidence of hospital-treated spinal trauma was 1 per 15,000 children [4].

Most studies on spinal injuries mainly include fractures, dislocations and unstable ligamentous injuries, usually confirmed with conventional radiographs or computed tomography (CT). Spinal magnetic resonance imaging (MRI) is usually recommended if cord injury is suspected [5], but the role of emergency MRI is controversial in the absence of neurological abnormalities. MRI is the most specific modality for soft tissue and craniocervical junction injuries and is essential for accurate diagnosis as there are no reliable clinical signs to rule them out [5, 6]. Although the role of MRI as a secondary modality after negative CT has been studied, the clinical feasibility and diagnostic reliability of first-line emergency MRI in pediatric spine trauma are poorly known. Lee et al. [7] recently published a retrospective analysis of 269 pediatric trauma patients undergoing cervical MRI in an emergency setting. They found that MRI is safe and 100% sensitive to unstable injuries and concluded that MRI should be considered an alternative to CT in pediatric spinal trauma. However, there is limited knowledge about using MRI as first-line imaging in pediatric spinal trauma.

Our institution has an MRI scanner dedicated only to emergency imaging. Therefore, we have been able to use MRI as the first-line imaging method in suspected low-impact spinal trauma. This retrospective study assessed the ability of MRI to exclude unstable injuries requiring surgical intervention when used as first-line emergency imaging in low-impact pediatric spinal trauma. We also examined the feasibility and safety of emergency MRI in this patient group.

## Materials and methods

Charts from patients who had undergone an emergency spinal MRI at our institution between April 1, 2013 and August 31, 2021 were retrospectively reviewed. Our institution is a tertiary care referral center for approximately 470,000 people. Permission from the hospital district board was obtained, but institutional review board approval and written patient consent were unnecessary due to the retrospective nature of the study. Inclusion criteria were (1) first-line emergency spinal MRI due to acute trauma, (2) age under 18 and (3) low-impact injury. A low-impact injury was defined as an injury not severe enough to trigger the standardized trauma team protocol [8]. Exclusion criteria were (1) severely altered consciousness, (2) unstable hemodynamics and (3) suspected child abuse.

The MRI scans were referred by an on-call physician, usually a pediatric orthopedic surgeon, trauma surgeon or neurosurgeon. In our institution, patients with low-impact trauma presenting worrisome symptoms such as altered mental state, neurological symptoms or severe pain are scanned immediately, preferably with MRI. However, CT is used if MRI is not instantly available or the patient is deemed not clinically suitable for a longer MRI scan.

 Our radiology information system (RIS) was reviewed to extract relevant information (radiology reports with MRI findings, seniority of the reporting radiologist, prior or complementary spinal imaging, follow-up imaging and concomitant brain imaging). MRI findings were first categorized into two groups: those associated with acute trauma and other findings. The acute traumatic findings were then categorized explicitly by location, extent of injury and type of injured structures. A retrospective radiological review of the imaging data was not performed because our primary goal was to study the clinical outcome of the patients who had undergone spinal MRI in a real-life setting. Medical records were reviewed for demographic and clinical variables: age, mechanism of injury, delay to admission, delay to imaging, need for anesthesia, concomitant injuries, treatment, follow-up and the final clinical outcome. To evaluate the justification for imaging in retrospect, we calculated Pediatric Emergency Care Applied Research Network (PECARN) risk scores for cervical trauma patients [5, 9].

MR imaging was performed in the emergency radiology department using a Philips Ingenia 3-tesla system with a Philips dStream coil system (Philips Healthcare, Best, Netherlands). The standard MRI protocol included sagittal T1-weighted, sagittal and axial T2-weighted and sagittal and coronal short tau inversion recovery (STIR) sequences. In selected cases, sagittal diffusion-weighted and sagittal gradient-echo T2*-weighted sequences were also acquired. The dedicated small field of view (FOV) proton density- and T2-weighted series were used for the craniocervical junction (occipital bone–second cervical vertebra, *C0–C2*) when needed. The detailed MRI parameters of the routine spine trauma protocols are described in Supplementary Material 1.

A large FOV was used to see the full extent of the injury. The cervical spine MRI was extended to cover the upper third of the thoracic spine and the lumbar MRI extended to cover the lower third of the thoracic spine.

Our standard clinical practice is to perform MRI without anesthesia or sedation whenever possible. The need for anesthesia was primarily assessed by referring physicians case by case; there were no definite rules regarding which age groups were sedated. The radiographers also requested a reassessment if the examination could not be performed without sedation.

The reference standard in this study was clinical outcome, primarily the need for surgical intervention. Information concerning clinical outcome was extracted from the medical records. When applicable, we sought the last appointment with the pediatric orthopedic surgeon. The total follow-up time was defined as follows: from the emergency MRI to the last date the patient resided in the municipality within our hospital district. Our hospital is the only center in this district that provides pediatric spinal surgery. Hence, in our healthcare system, assuming that late-onset problems demanding surgical attention would have emerged in the medical records is justified. Patients with less than 6 months of follow-up were excluded from the assessment of clinical outcomes. Feasibility of the emergency MRI was assessed by the need for anesthesia to conduct the examination, the proportion of images performed successfully on the same day and MRI artifacts; safety was assessed by MRI- or anesthesia-related adverse events.

The results are expressed as the number of cases (*n*), percentage, median, mean and standard deviation (SD). The normality of probability distributions was tested using the Kolmogorov–Smirnov and Shapiro–Wilk tests. The Mann–Whitney *U* test was used to compare means for non-normally distributed variables. Proportions of categorical variables were compared with the Pearson Chi-square (Χ^2^) test. One-way ANOVA was used to compare the means of multiple groups. *P*-values < 0.05 were considered statistically significant. The statistical analyses were performed using IBM SPSS Statistic s Package for Mac (version 28, IBM Corporation, Armonk, NY).

## Results

A total of 396 patients met the inclusion criteria. The mean age was 11.5 years and the median was 12.0 years (range 0–17). Acute trauma findings were detected in 114 (29%) scans. Table 1 represents the study population’s demographic characteristics, injury mechanisms, follow-up data and clinical outcomes. Falling was the most common cause of injury among the whole group, accounting for 28% of cases (109/396). Among those with traumatic MRI findings (114 patients), trampoline accidents were the leading cause, at 32% (36/114).

**Table 1:** Characteristics of the study population, types of injury and outcome

	Total	MRI positive	MRI negative	*P*-value
Number of cases	396	114	282	
Age, mean (SD)	11.5 (3.6)	11.0 (3.2)	11.7 (3.7)	0.021
Age, median (range)	12 (0–17)	11 (2–17)	12 (0–17)	
Male, *n* (%)	181 (45.8)	60 (52.6)	122 (43.3)	0.121
Mechanism of injury, *n* (%)				
Fall	109 (27.5)	26 (22.8)	83 (29.4)	0.267
Trampoline	69 (17.4)	36 (31.6)	33 (11.7)	< 0.001
Contact sports	50 (12.6)	8 (7.0)	42 (14.9)	0.031
Gymnastics	32 (8.1)	13 (11.4)	19 (6.7)	0.155
Horseback riding	29 (7.3)	7 (6.1)	22 (7.8)	0.673
Moped, all-terrain vehicle	26 (6.6)	2 (1.8)	24 (8.5)	0.013
Winter sports	18 (4.5)	7 (6.1)	11 (3.9)	0.425
Violence by another child	18 (4.5)	4 (3.5)	14 (5.0)	0.605
Motor vehicle accident	14 (3.5)	4 (3.5)	10 (3.5)	1.000
Pedestrian struck by car	3 (0.8)	1 (0.9)	2 (0.7)	1.000
Other^a^	28 (7.1)	6 (5.3)	22 (7.8)	0.398
Follow-up				
Follow-up appointment with pediatric orthopedic surgeon	108 (27.3)	79 (69.3)	29 (10.3)	
Last appointment with a pediatric orthopedic surgeon, median, weeks after emergency MRI (range)	6 (1–110)	6 (1–110)	2 (1–104)^b^	
Total follow-up period, median, months after emergency MRI (range)	41 (0–98)	41 (0–98)	41 (0–98)	
Patients with a follow-up of ≥ 6 months, *n* (%)	376 (94.9)	107 (91.5)	269 (96.4)	
Outcome in patients with a follow-up of ≥ 6 months, *n* (%)				
No permanent consequences	358 (95.2)	106 (93.0)	260 (96.7)	0.112
Prolonged pain	16 (4.3)	7 (6.5)	9 (3.3)	0.256
Postoperative junctional kyphosis	1 (0.3)	1 (0.9)	-	0.287

*MRI* magnetic resonance imaging, *SD* standard deviation

^a^Including bicycle/kick scooter, diving, accidental hit in the head

^b^One patient with a 104-week follow-up by the pediatric orthopedic surgeon was treated for non-traumatic spondylolysis found incidentally on the emergency magnetic resonance imaging study

Of the patients undergoing cervical spine MRI, 93% (289/310) had at least one PECARN risk factor and of the patients with thoracolumbar MRI, 92% (79/86) had symptoms suggesting thoracolumbar injury. None of the patients was unconscious. The Glasgow Coma Scale was 14–15 in 98% (388/396) and 11–13 in 2% (8/396) of patients (Supplementary Material 2).

Our study population had no deaths or permanent neurological deficits (Table 1). None of the patients with negative spinal MRI after acute injury required surgery or immobilization, suggesting a negative predictive value of 100%. There were 9 cases (9/282, 3%) in the MRI-negative group with prolonged non-specific pain after the injury, including two patients with concomitant brain injury.

**Fig. 1:**
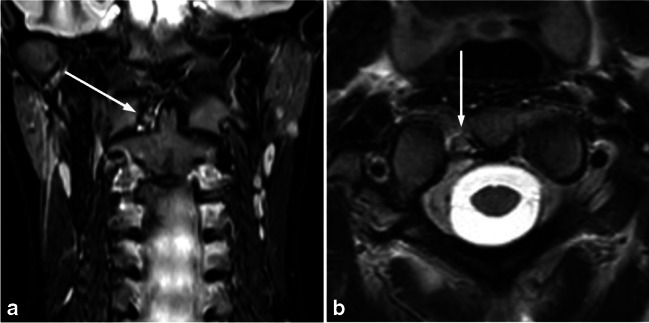
Partial tear of the right transverse ligament (*arrows*) in a 14-year-old girl after a basketball accident. **a** Coronal short tau inversion recovery magnetic resonance image (MRI). **b** Axial T2-weighted MRI

**Fig. 2:**
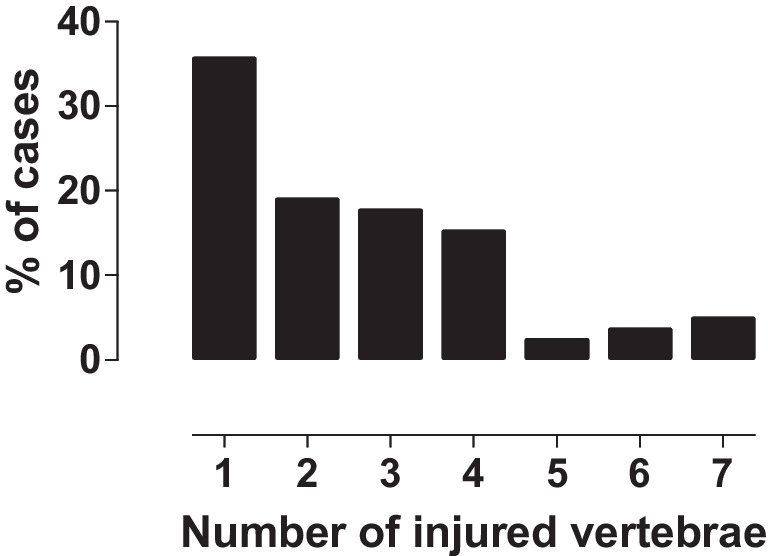
Patients with bony injury. Percentage of cases with different numbers of injured vertebrae

Detailed information concerning trauma findings in the MRI examinations is presented in Tables 2–3. The bony vertebrae were the most commonly injured structure (78/114, 68%), followed by ligaments (Table 2). The ligamentous injuries occurred most often in the interspinous ligament. There were 22 cases with isolated injuries of the interspinous ligament (22/114, 19%) and 11 cases with interspinous ligament injury combined with injury of the ligamentum flavum, the anterior longitudinal ligament or the transverse ligament (11/114, 10%). Ligamentous injuries occurred at the C0–C2 level in 6/114 (5%) (Fig. 1). Of the patients with traumatic MRI findings, 38/114 (33%) had at least two different types of injury. The most common level of injury was the thoracic spine (33/114, 29%), followed by the subaxial cervical spine and combined injury of more than one level (both 25/114, 22%). Of all individuals with traumatic findings on MRI, 27/114 (24%) had a noncontiguous injury with one or more spared vertebral levels between injuries (Table 3). A total of 50/114 (64%) individuals with acute bony injury had a fracture or a bone contusion of more than one vertebra, with a maximum of seven separate vertebrae involved (Fig. 2).

**Table 2:** Characteristics of traumatic findings on magnetic resonance imaging

	*n* (%)
Bony	78 (68.4)
Ligamentous (any ligament)	41 (36.0)
Interspinous	22 (19.3)
Interspinous and flavum	7 (6.1)
Alar	4 (3.5)
Transverse	2 (1.8)
Interspinous and flavum and nuchae	2 (1.8)
Flavum, ALL and interspinous, transverse and flavum and interspinous, nuchae (one each)	4 (3.6)
Cord injury	-
Epidural hematoma	1 (0.9)
AARF/AARS	2 (1.8)
Traumatic spondylolisthesis	2 (1.8)
Facet or uncovertebral joint injury	8 (7.0)
Intervertebral disc	3 (2.6)
Muscle	26 (22.8)
Paraspinal	10 (8.8)
Suboccipital	6 (5.3)
Paraspinal and suboccipital	1 (0.9)
Miscellaneous	9 (7.9)
Other (nerve root, sternum, abdomen)	3 (2.6)
Soft tissue edema only	10 (8.8)
Multiple injury types	
Yes	38 (33.3)
No	76 (66.7)

*ALL* anterior longitudinal ligament, *AARF/AARS* atlantoaxial rotatory fixation/atlantoaxial rotatory subluxation

**Table 3:** Level of traumatic findings on magnetic resonance imaging

Injured levels	*n* (%)
C0–C2	11 (9.6)
Subaxial cervical spine	25 (21.9)
Thoracic spine	33 (28.9)
Lumbar spine	12 (10.5)
Sacral spine	8 (7.0)
Combined	25 (21.9)
C0-C2 + subaxial	3 (2.6)
C0-C2 + thoracic	1 (0.9)
C0-C2 + subaxial + thoracic	4 (3.5)
Subaxial + thoracic	12 (10.5)
Subaxial + thoracic + lumbar	1 (0.9)
Thoracic + lumbar	2 (1.1)
Thoracic + sacral	1 (0.9)
Thoracic + lumbar + sacral	1 (0.9)
Lumbar + sacral	2 (1.8)
Contiguous injury	
Yes	87 (76.3)
No	27 (23.7)

*C0* occipital bone, *C2* second cervical vertebra

**Fig. 3:**
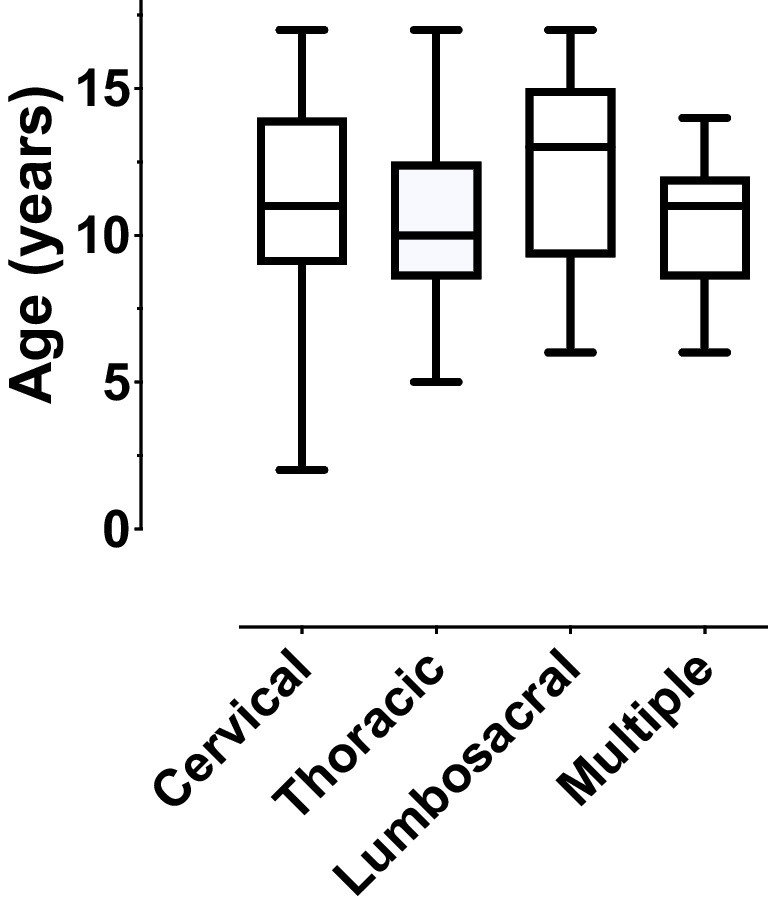
Level of spine injury plotted against patient age

**Fig. 4:**
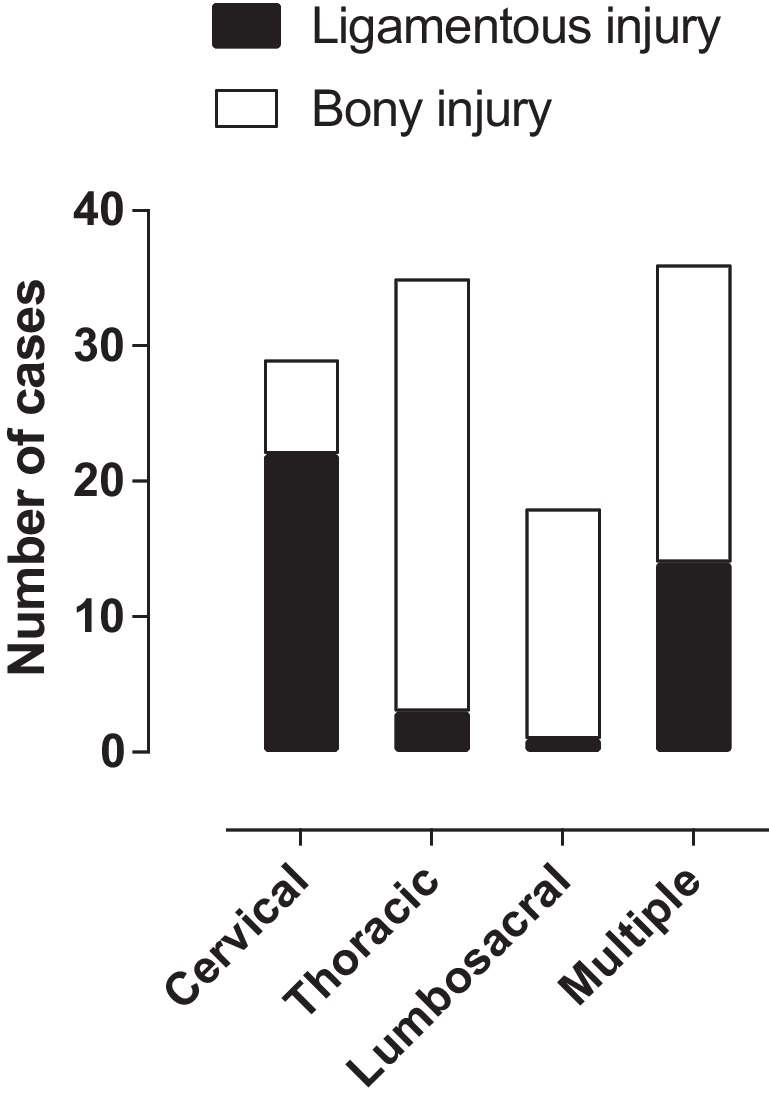
Distribution of injured structures at different levels of the spine. Ligamentous injuries were more common in the cervical spine and bony injuries in the thoracolumbosacral spine

Age was not statistically significantly associated with the injured spinal level (one-way ANOVA, *P*=0.190, Fig. 3). The prevalence of ligamentous injuries was higher in the cervical spine than in the subcervical spine (Χ^2^ = 33.2, *P* < 0.001), whereas the reverse was found for the prevalence of bony injuries (Χ^2^ = 59.4, *P* < 0.001 for bony injuries) (Fig. 4).

Only 3/396 (0.8%) of the study population required surgery and none needed halo bracing. Supportive therapy only (analgesics, activity restriction or soft collar without strict immobilization) was given to 33/114 (29%) patients with MRI findings and 13/114 patients (11%) with minor findings did not need any specific treatment. Supportive therapy was also used for some patients without any trauma findings on MRI (Table 4). The total follow-up time was 6 months or more for 376 (95%) patients (median age 12 years, range 0–17 years). At least one pediatric orthopedic surgeon follow-up appointment was arranged for 108 (27%) patients (the median time from MRI to appointment was 6 weeks, ranging from 1 to 110 weeks). After the follow-up period determined by the responsible physician, none of the patients required surgical treatment.

**Table 4:** Treatment used in the study population

Treatment	*n* (%)
Any immobilization	84 (21.2)
Rigid cervical collar	51 (12.9)
Extension brace	25 (6.3)
Glisson’s traction	5 (1.3)
Surgery	3 (2.6)
Halo brace	-
Analgesics	165 (41.7)
Activity restriction	102 (25.8)
Soft collar	27 (6.8)
Supportive therapy only (analgesics, soft collar or activity restriction without immobilization)	141 (35.6)

We were able to scan 95% of the patients without sedation or anesthesia (Supplementary Material 3), mainly those aged 5 years or older. Of all the patients included in the study, 16/396 (4%) were scanned following sedation (thiopental, dexmedetomidine, propofol or combination) with spontaneous breathing and only 3/396 (0.8%) required general anesthesia with intubation. No immediate complications or adverse events related to sedation or general anesthesia occurred.

Table 5 shows that we were able to scan 95% of the patients on admission or the next day. We had to suspend one scan (1/396, 0.3%) before obtaining diagnostic images due to insufficient patient cooperation, but the scan was successfully performed the next day. All other scans were performed with adequate diagnostic image quality at the first attempt.

**Table 5:** Intervals from injury to admission and magnetic resonance imaging

Days	MRI positive			MRI negative		
	Injury to admission, *n* (%)	Admission to MRI, *n* (%)	Injury to MRI, *n* (%)	Injury to admission, *n* (%)	Admission to MRI, *n* (%)	Injury to MRI *n*, (%)
0	90 (78.9)	103 (90.4)	80 (70.2)	217 (77.0)	239 (84.8)	181 (64.2)
1	12 (10.5)	9 (7.9)	19	33 (11.7)	26 (9.2)	56 (19.9)
2–7	10 (8.8)	2 (1.8)^a^	12 (10.5)	24 (8.5)	15 (5.3)	37 (13.1)
8–23	2 (1.8)	-	3 (2.6)	8 (2.8)	2 (0.7)	8 (2.8)
Mean, days (SD)	0.8 (2.6)	0.1 (0.4)	0.9 (2.6)	0.7 (1.9)	0.4 (1.6)	1.0 (2.5)

*MRI* magnetic resonance imaging, *SD* standard deviation

^a^Two and 3 days

Additional CT imaging was performed for 15 patients (4%) in the emergency department. All complementary CTs were suggested by a radiologist on call, primarily to further examine suspected or confirmed bony injuries; one CT was performed because of motion artifacts on some of that patient's MRI sequences. None of the complementary CTs revealed injuries not seen in MRI (Table 6). In the 15 patients with both emergency MRI and complementary CT, the sensitivity and specificity of MRI were 100% and 23%, respectively. In total, MRI artifacts were mentioned in five reports (5/396, 1.3%), three minor without warranting additional imaging.

**Table 6:** Patients with additional computed tomography imaging

Age (years)	FOV	Indication	Additional information on CT	Conclusion
6	C0–Th3	Motion artifacts in some sequences, complementary imaging	None	No trauma
7	C0–C3 rotational CT	Dens asymmetry, no ligamentous injury or bone marrow edema	None	No trauma
8	C3–C7	Vertebral body edema	No anatomical compression	Bone contusion
9	C5–C7	Vertebral body edema	No anatomical compression	Bone contusion
9	C0–C3	Torticollis, MRI negative	None	No trauma
9	Low-dose thoracic CT	Suspected sternal fracture	No sternal fracture	Thoracic vertebral fracture seen on MRI was not visible on CT
10	L2	Vertebral body fracture	None	Fracture, just as seen in MRI
10	C3–C5	Artifact-like signal on C4, the fracture could not be excluded	No fracture	No trauma
10	C0–C6	Suspected facet joint fracture	No fracture	Flavum injury, interspinous injury, facet capsule injury on MRI
11	L4–S1	Non-traumatic spondylolysis	Spondylolysis was thought to be chronic, but not pseudoarthrotic	No trauma
11	C3–Th1	Suspected facet joint fracture	No fracture	Facet joint subluxation, flavum injury, interspinous ligament injury on MRI
11	C2–C5	Uncovertebral joint effusion	No fracture	No trauma
12	C0–T3	Suspected facet joint fracture	No fracture	PLC injury and facet joint subluxation on MRI
15	C4–C7	C6 bone marrow edema	No anatomical compression/fracture	Bone contusion
17	C4–T3	Vertebral body edema, suspected fracture	No anatomical compression	Bone contusion
Sensitivity and specificity of MRI in spinal fractures when using targeted CT as a reference standard (*n*=15)				
Sensitivity			1.00	
Specificity			0.23	
Positive predictive value			0.17	
Negative predictive value			1.00	

*C0* occipital bone, *C2* second cervical vertebra, *C3* third cervical vertebra, *C4* fourth cervical vertebra, *C5* fifth cervical vertebra, *C6* sixth cervical vertebra, *C7* seventh cervical vertebra, *CT* computed tomography, *L2* second lumbar vertebra, *MRI* magnetic resonance imaging, *PLC* posterior ligament complex, *S1* first sacral vertebra, *T1* first thoracic vertebra, *T3* third thoracic vertebra

Of 396 MRI reports, 2 (0.5%) were written by a radiology resident (> 3 years of experience in radiology) and 394 (99.5%) were written by a board-certified radiologist (> 5 years of experience in radiology), including 378 (95.4%) by fellowship-trained neuroradiologists, musculoskeletal radiologists or emergency radiologists (> 7 years of experience in radiology). All 15 CT reports were written by board-certified radiologists (> 5 years of experience in radiology), including 13 (87%) fellowship-trained neuroradiologists, musculoskeletal radiologists, emergency radiologists, or pediatric radiologists (> 7 years of experience in radiology). Standardized reports on spinal MRI were not used.

## Discussion

In our study of a large sample of pediatric patients with low-impact spinal trauma who underwent emergency MRI scans as the first-line imaging, we found an excellent ability of MRI to exclude injuries requiring surgical treatment. No missed injuries were found during the clinical follow-up, most patients were scanned without anesthesia and no MRI-related adverse events occurred.

Imaging is crucial in diagnosing pediatric spine injuries, but the most suitable imaging modality, especially after a low-impact trauma, is still debatable. Patients with high-impact trauma are usually examined acutely with whole-body trauma CT including thoracolumbosacral and cervical spine scans [8]. Also, when excluding trauma protocols, conventional radiographs and CT are widely recommended [5, 6]. The role of MRI in the diagnostic workup of symptomatic patients with negative CT or low-impact trauma has been controversial. Qualls et al. [10] and Derderian et al. [11] did not find MRI to be useful in addition to CT in detecting unstable cervical spine injuries and Franklin III et al. [12] stated that adding MRI to thoracolumbar fractures shown by CT did not change treatment or outcome. Moore [13] found MRI to be more sensitive and specific than conventional radiographs and concluded that conventional radiographs might not be justified in clearing the pediatric cervical spine due to low negative predictive value on symptomatic but non-obtunded patients. A meta-analysis by Schoenfeld et al. [14] and a recent paper by Al-Sarheed et al. [15] concluded that MRI is needed to clear the cervical spine of an unconscious or unexaminable child, even if a cervical spine CT reveals no sign of trauma.

Henry et al. [16] compared the specificity and sensitivity of MRI and CT in pediatric cervical trauma. MRI was considered a standard for ligamentous/soft tissue injury and CT for bony injury. MRI was almost as good as CT in detecting osseous injuries and far superior for soft tissue/ligamentous injuries. The authors suggested that MRI could also serve as a screening tool for bony injuries. Another study by Henry et al. [17] showed that STIR MRI had good sensitivity in pediatric cervical spine injuries and may be of clinical use in the clearance of the pediatric cervical spine. In a recent study by Lee et al. [7], MRI was found to be 100% sensitive to unstable cervical injuries.

Our results may not be generalizable for pediatric patients with high-energy trauma, but they do support previous findings of the excellent accuracy of MRI in ruling out unstable spinal injury. We did not systematically examine the efficacy of individual MRI sequences. However, in concordance with Henry’s work [17], our practical experience suggests that in most cases, STIR is the most valuable sequence, with other sequences being complementary and confirmatory. A short screening MRI protocol including only STIR might be possible in selected cases, but this should be prospectively evaluated. The shortened protocol might help integrate MRI into standard clinical practice more widely.

In our cohort, ligamentous injuries were more common in the cervical spine and bony injuries in the thoracolumbosacral spine. Of all patients with traumatic findings on MRI, 25/114 (21.9%) had injuries affecting more than one level of the spine, suggesting the need for a sufficiently large FOV for imaging. We routinely extend the imaging of the cervical spine to the upper third of the thoracic spine.

Exposure to ionizing radiation and its long-term effects is a constant issue in pediatric imaging. The lifetime risk of malignancy-related medical imaging in the population is quantifiable although small [18, 19]. The consequences of missed spinal injury can be devastating and radiation exposure is usually justified. MRI has the advantage of lack of ionizing radiation but also disadvantages, including lower availability, higher cost and longer scanning times, often requiring anesthesia or sedation for younger children to achieve adequate image quality [20, 21]. Nevertheless, the higher cost of MRI scans could be mitigated by shorter intensive care treatment and hospital stays [22].

Small children are thought to be prone to cervical spine injuries. However, we did not find significant differences between injured levels in different age groups (Fig. 3). One explanation might be that, as mentioned earlier, we saw many soft tissue injuries and bone contusions that could have been nondetectable on radiographs or CT. The difference in the incidence of ligamentous and bony injuries was statistically significant at different spine levels (Fig. 4): ligamentous injuries were more common in the cervical spine and bony injuries were more common in the thoracolumbar spine.

**Fig. 5:**
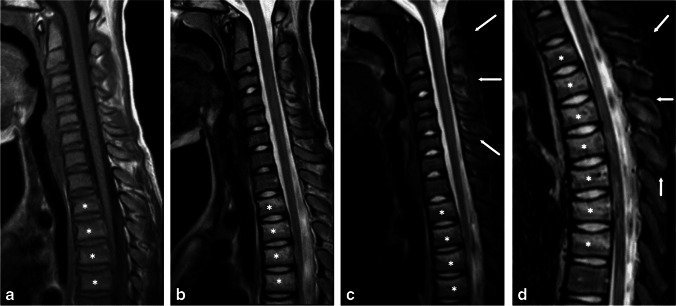
Magnetic resonance images of an 11-year-old boy after a trampoline accident. **a**–**c** Sagittal T1 (**a**), T2 (**b**) and short tau inversion recovery (STIR) (**c**) of the cervical spine. **d** Sagittal STIR of the thoracic spine show compression fractures of the second to eighth thoracic vertebrae (*asterisks*) and edema in the interspinous ligament (*arrows*)

**Fig. 6:**
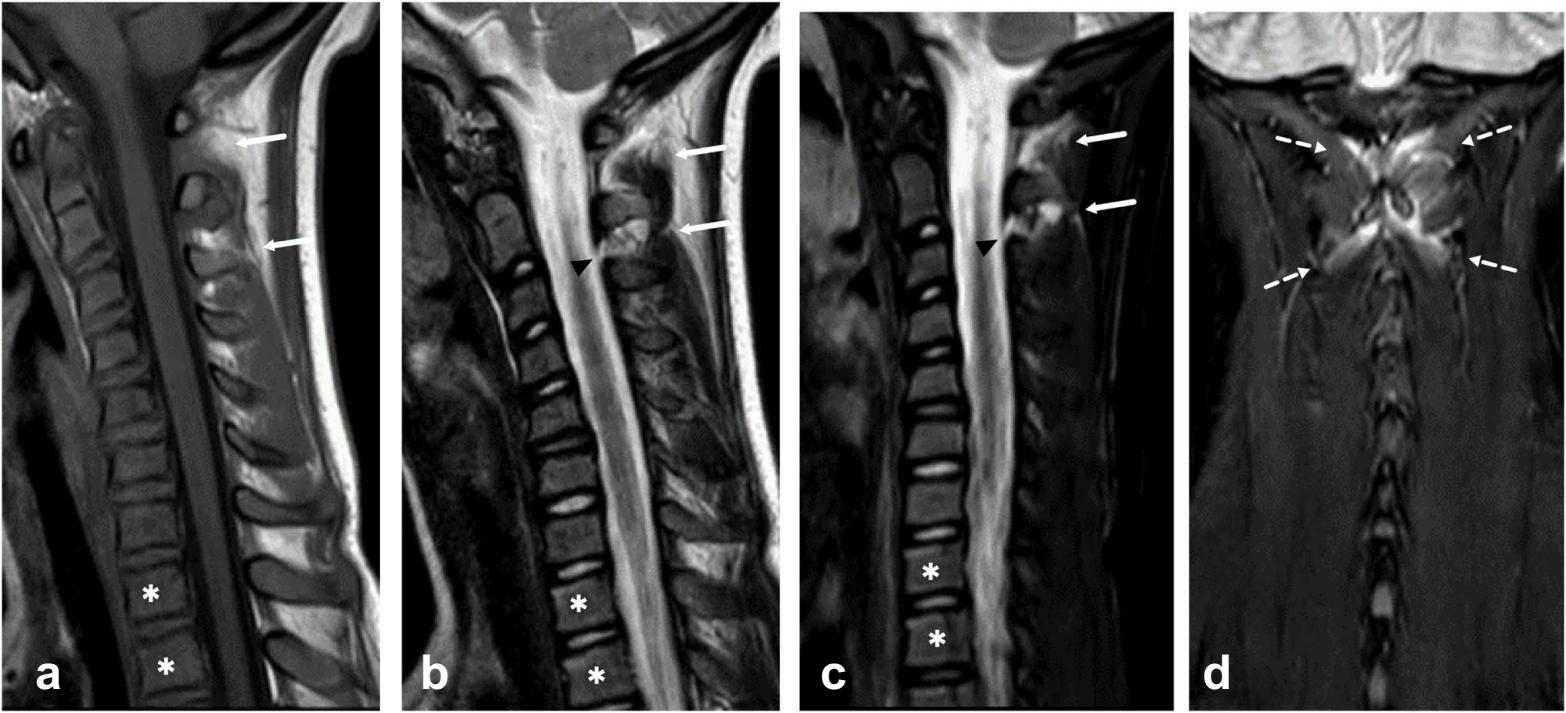
Images of a 10-year-old girl after a trampoline accident. Sagittal T1-weighted (**a**), sagittal T2-weighted (**b**), sagittal short tau inversion recovery (STIR) (**c**) and coronal STIR magnetic resonance images show posterior ligament complex injury at the cervical (C)1–C3 levels and minor compression fractures of thoracic vertebral bodies 1 and 2 (*asterisks*). The ligamentum flavum (*arrowheads*) is torn at C2/C3 and the interspinous and supraspinous ligaments (*arrows*, the two ligaments cannot be identified separately in these images) are torn at C1/C2 and C2/C3 levels. The suboccipital muscles are edematous (*broken arrows*)

A remarkable number (50/78, 64%) of patients with bony injuries had traumatic MRI findings of more than one vertebra (Fig. 2, Fig. 5, Fig. 6). In addition, almost a quarter (27/114, 24%) of the patients with traumatic findings had noncontiguous injuries (Table 3, Fig. 5, Fig. 6). These findings suggest that imaging should have wide coverage of the spine and not just the most suspicious level. This makes MRI without ionizing radiation an even more attractive option.

**Fig. 7:**
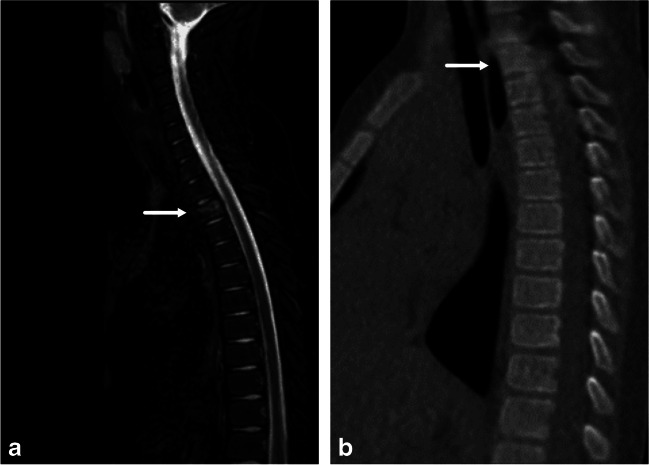
Compression fracture (*arrows*) of the third thoracic vertebra in a 9-year-old boy who fell from a swing. A low-dose computed tomography (CT) scan was performed to exclude a suspected sternal fracture. The vertebral fracture was not visible on CT scan. **a** Sagittal short tau inversion recovery magnetic resonance image. **b** Low-dose non-contrast-enhanced thoracic CT, sagittal reformat

Immediate additional CT imaging was obtained in 15 children (Table 6). All CT scans were suggested by a radiologist on call to further investigate a suspected fracture or joint dislocation. Only 1/15 additional spinal CT scan covered the entire cervical spine. The others were targeted to a specific suspicious segment. In total, five complementary CT scans helped rule out spinal trauma and eight were concordant with MRI, showing no further bony injuries. Thoracic CT was performed in one patient because of a suspected sternal fracture on MRI. No sternal fracture was found and the vertebral contusion seen on MRI was not visible on CT (Fig. 7). When the sensitivity and specificity were calculated using targeted CT as a reference standard, MRI was 100% sensitive and 23% specific. This reflects the ability of MRI to reveal bone bruises not visible on CT. Although only limited conclusions can be drawn from 15 patients, this finding underlines the potential pitfall of interpreting bone bruises as fractures. If no fracture line or vertebral height loss is seen, the term “fracture” should not be used. The specificity of MRI for bony injuries can be improved with zero echo time imaging (ZTE) with significantly better visualization of detailed bony structures compared to T1-weighted sequences [23, 24], reducing the need for CT. We did not use the ZTE technique in this study.

Most patients were scanned on the day of admission: 90% in the group with findings on MRI and 85% in the group without traumatic findings. In our institution, patients with more alarming symptoms such as altered mental state or severe pain are always scanned immediately, either with MRI or CT. In the population with milder symptoms, it is possible, albeit not ideal, to postpone imaging without considerable risk if MRI is not immediately available. The interval between injury and admission was longer in the group with negative MRI, consistent with milder symptoms and presumably lower probability of significant injury. Still, over 90% of our patients had symptoms that, according to the American College of Radiology (ACR) Appropriateness Criteria [5], should lead to spinal imaging (Supplementary Material 2).

Only 2.6% (3/114) of the patients with findings on MRI and 0.8% (3/396) of the entire cohort needed surgical treatment. This reflects the low-impact trauma of our study population, with high-impact injuries being assessed by a trauma team and initial CT imaging. Patients with prior spinal CT were excluded from this study, although many were referred to spinal MRI after stabilizing their critical conditions. Either way, the proportion of surgically treated patients in the study population is very low, signaling an unnecessarily low threshold for performing imaging in this patient group. As 93% of the patients with cervical spine MRI fulfilled the ACR Appropriateness Criteria for spinal imaging, our results suggest that the PECARN score has excellent sensitivity but low specificity. The low percentage of severely injured patients in our study population concords with the retrospective study of Phillips et al. [25]. They highlighted that the use of contemporary clinical decision-making rules has probably increased imaging rates, even though the aim of these rules is the opposite. The need for more accurate clinical decision-making tools is apparent.

All three operatively-treated injuries were cervical injuries after a trampoline accident (Fig. 6). In the group with at least 6 months of clinical follow-up, prolonged pain was reported in 6.5% of the cases in the group with traumatic MRI findings and 3.3% in the group with no findings on MRI. None of the children with prolonged pain needed to adjust their everyday life due to back pain. To our knowledge, no published data exist on the normal incidence or prevalence of prolonged post-traumatic spinal pain in children. Spinal pain is generally relatively common among children and adolescents. For example, in a Danish school-based prospective cohort study, 14–20% of the minors aged 11–15 years reported frequent spinal pain [26]. Therefore, it seems unlikely that the cases with prolonged pain are due to injuries missed on emergency MRI. Perhaps, on the contrary, a negative emergency MRI scan might reassure children and their parents.

We observed stable soft tissue injuries, bone contusions and other injuries that do not need surgical treatment or immobilization or whose treatment is not yet fully established (Fig. 5). MRI can be criticized for being too sensitive, leading to unnecessary use of collar or follow-up imaging. Possible overtreatment is an important issue and clinical treatment protocols should be adjusted to account for more information available from these patients when using MRI scanning. These subtle findings are also valuable for children and their families because they explain the pain and other symptoms. Informing patients and their families about the injuries may reduce future healthcare contacts and decrease expenditure.

No MRI- or anesthesia-related adverse effects were reported in this study population, consistent with previously published observations of an excellent safety profile of MRI [7]. Our study shows that in a selected pediatric population, spinal MRI can usually be obtained without sedation or anesthesia for children aged 5 years or older (Supplementary Material 3). Of patients scanned under anesthesia, 16 (4%) were sedated with spontaneous breathing and only three (0.8%) were intubated. However, our cohort may be biased as the patients might have been referred to MRI due to expected sufficient cooperation without the need for anesthesia.

Our study has limitations, most importantly due to its retrospective design. Another notable limitation is the lack of systematic comparison between MRI and CT or conventional radiographs. Nevertheless, considering the excellent patient outcome in our study population, exposing children to ionizing radiation to conduct a comparative study is difficult to justify. Previous studies have established the yield of follow-up MRI after negative CT [6, 14, 15]. Our study population might be biased because not all pediatric low-impact trauma patients were imaged with MRI. Some cases with more worrisome symptoms might have undergone CT if the MRI was not immediately available. Another issue limiting the generalizability of our results is the level of experience among the radiologists reporting the MRI examinations included in the study (95% by fellowship-trained subspecialists), as not all emergency departments may have on-call radiologists with similar competence. We feel that these limitations are unlikely to bias our results significantly. Nevertheless, more studies and education are needed to extensively implement the first-line use of MRI in pediatric spinal trauma imaging. 

Most importantly, we found the clinical outcome of the patients in our study cohort to be excellent (Table 1). None of the 376 patients with at least 6 months of follow-up was found to have injuries requiring surgery that were missed on the emergency MRI; no patient needed to be operated on or immobilized after the end of the follow-up period, determined by the responsible physician. Despite being low-impact trauma patients, our study population had indications for the spinal imaging [5] (Supplementary Material 2). In this well-known and relatively abundant group of patients suspected of spinal injury without high-impact trauma, MRI appears to help rule out spinal trauma without exposing children to ionizing radiation.

## Conclusion

We found MRI suitable for excluding injuries requiring surgical intervention when used as a first-line imaging method in suspected low-impact pediatric spinal trauma. No clinically significant injuries were missed based on clinical follow-up in the 376 patients with at least 6 months of follow-up. Most children aged 5 years or older were scanned without anesthesia and no MRI-related adverse events were reported. We conclude that MRI can be used as a first-line imaging modality in clearing the spine in the pediatric population with low-impact trauma.

### Supplementary Information

Below is the link to the electronic supplementary material.

Supplementary file1 (DOCX 18.7 kb)

## Data Availability

Data cannot be publicly shared because of national legislature on the privacy of patient data.
