# Supplementary material for: Clinical outcome following magnetic resonance imaging as first-line imaging in low-impact pediatric spine trauma: a single-center retrospective observational study
Source: Pediatr Radiol. 2023 Jul 31;53(11):2269–80. doi: 10.1007/s00247-023-05721-7 (PMC10562339; doi:10.1007/s00247-023-05721-7)
Supplement: Supplementary file 1 — Supplementary file1 (DOCX 18.7 kb) [file 247_2023_5721_MOESM1_ESM.docx]

**Supplementary Material 1 Sequence parameters in routine emergency magnetic resonance imaging**

|  | Cervical spine | | | | | Thoracolumbar spine | | | | | Craniocervical junction | | |
| --- | --- | --- | --- | --- | --- | --- | --- | --- | --- | --- | --- | --- | --- |
| Parameter | Sagittal T1 | Sagittal T2 | Sagittal STIR | Coronal STIR | Axial T2 | Sagittal T1 | Sagittal T2 | Sagittal STIR | Coronal STIR | Axial T2 | Sagittal PD | Coronal PD | Axial T2 |
| TR (ms) | 550 | 3584 | 3305 | 3721 | 5717 | 683 | 4955 | 4391 | 5778 | 3601 | 2500 | 2500 | 5902 |
| TE (ms) | 7 | 100 | 60 | 60 | 95 | 8 | 100 | 60 | 60 | 100 | 20 | 20 | 80 |
| Flip angle | 80 | 90 | 90 | 90 | 90 | 80 | 90 | 90 | 90 | 90 | 90 | 90 | 90 |
| Slice thickness (mm) | 3 | 3 | 3 | 3 | 3 | 3 | 3 | 3 | 4 | 3 | 2.5 | 2.5 | 2.5 |
| Slice spacing | 3.3 | 3.3 | 3.6 | 3.6 | 3.3 | 3.3 | 3.3 | 3.3 | 4.4 | 3.3 | 2.75 | 2.75 | 2.75 |
| Number of slices | 15 | 15 | 15 | 18 | 35 | 21 | 21 | 19 | 20 | 51 | 22 | 16 | 27 |
| Matrix size | 480 x 480 | 448 x 448 | 480 x 480 | 432 x 432 | 320 x 320 | 384 x 384 | 432 x 432 | 512 x 512 | 432 x 432 | 320 x 320 | 400 x 400 | 320 x 320 | 320 x 320 |

*PD* Proton density, *STIR* Short tau inversion recovery, *TE* Time to echo, *TR* Repetition time

**Supplementary Material 2 Quantity of Pediatric Emergency Care Applied Research Network cervical spine injury risk factors, thoracolumbar pain and Glasgow Coma Scale in the study population**

| PECARN C-spine risk factors (cervical spine MRI) | 0 | 1 | 2 | Total |
| --- | --- | --- | --- | --- |
| Number of cases with cervical spine MRI (%) | 21 (6.8) | 248 (80) | 41 (13.2) | 310 (100) |
| Thoracolumbar symptoms (thoracolumbar spine MRI)^a^ | No | Yes |  | |
| Number of cases with thoracolumbar spine MRI (%) | 7 (8.1)^b^ | 79 (91.9) |  | 86 (100) |
| GCS at the emergency | 14–15 | 11–13 | <11 |  |
| Number of cases (%) | 388 (98.0) | 8 (2.0) | 0 | 396 (100) |

*C-spine* Cervical spine, *GCS* Glasgow coma scale, *MRI* Magnetic resonance imaging, *PECARN* Pediatric Emergency Care Applied Research Network

^a^Pain, reported neurological abnormalities

^b^The whole spine was scanned, but the highest suspicion of injury was in the cervical spine

**Supplementary Material 3 The need for anesthesia**

| Need for anesthesia | *n* (%) | Mean (yr) | SD | Min | Max |
| --- | --- | --- | --- | --- | --- |
| No | 377 (95.2) | 11.9 | 3.1 | 4 | 17 |
| Yes | 19 (4.8) | 3.5 | 2.8 | 0 | 10^a^ |

*SD* Standard deviation

^a^Of nineteen patients scanned in anesthesia, three were older than five years: 7- and 9-year-old patients had a concomitant traumatic brain injury and a 10-year-old patient had a neuropsychiatric disorder
